# Preoperative Anxiety in Greek Children and Their Parents When Presenting for Routine Surgery

**DOI:** 10.1155/2018/5135203

**Published:** 2018-07-02

**Authors:** Aikaterini Charana, Gregory Tripsianis, Vasiliki Matziou, Georgios Vaos, Christos Iatrou, Pelagia Chloropoulou

**Affiliations:** ^1^University Hospital of Alexandroupolis, Democritus University of Thrace, Alexandroupolis, Greece; ^2^Department of Medical Statistics, Medical School, Democritus University of Thrace, Alexandroupolis, Greece; ^3^Faculty of Nursing, National and Kapodistrian University of Athens, Athens, Greece; ^4^Department of Paediatric Surgery, Atikkon University General Hospital, Medical School, National and Kapodistrian University of Athens, Athens, Greece; ^5^Department of Anesthesiology, Medical School, Democritus University of Thrace, Alexandroupolis, Greece; ^6^Department of Anesthesiology, University Hospital of Alexandroupolis, Alexandroupoli, Greece

## Abstract

**Background:**

A surgical operation in pediatric patients is a rather stressful experience for both children and their parents. The aim of this study was to assess the effect of specific demographic characteristics in parent's and children's preoperative anxiety.

**Methods:**

The sample was composed of 128 Greek-speaking children (1–14 years of age) who had to undergo minor surgery in a University General Hospital. Before surgical operation, the Spielberger State-Trait Anxiety Inventory (STAI) questionnaire and a questionnaire for the social-demographic characteristics were completed by the parents. Children's preoperative anxiety was evaluated using the Modified Yale Preoperative Anxiety Scale (m-YPAS).

**Results:**

The independent predictors of increased anxiety levels in parents are child's age (*p*=0.024) and gender (girls: *p*=0.008), living in rural areas (parents: *p* < 0.001; children: *p*=0.009), being a mother (*p*=0.046), high or low education level (*p*=0.031), a no premedicated child (*p*=0.007), and high baseline parental anxiety (*p*=0.003). Previous hospitalization (*p*=0.019), high situational parental anxiety (*p* < 0.001), no premedication (*p*=0.014), and being the only child in the family (*p*=0.045) are found to be the main determinants of preoperative anxiety control in children.

**Conclusions:**

This study identifies possible risk factors of preoperative anxiety in parents and their children, which are high parental anxiety, child's age, no premedication, being the only child in the family, living in rural areas, education level, and previous hospitalization.

## 1. Introduction

A scheduled surgical operation in a child is a rather stressful procedure not only for the child but also for the whole family. Surgery in pediatric patients is an unpleasant and potentially “threatening” experience which is usually followed by preoperative anxiety due to the child's illness, hospitalization, fear of anesthesia—especially the time of induction of anesthesia—and the operation itself [1]. Moreover, it is well established that preoperative anxiety in children may lead to negative postoperative responses, including longer hospitalization period, more pain, and long-term behavioral problems [2, 3].

Premedication, usually oral or intranasal midazolam, is used routinely to treat anxiety in children with several studies supporting its benefits [4, 5]. On the other hand, there are studies that argue against the use of premedication because of its possible side effects and extra cost [6, 7]. As a result, there are other drugs that have been studied in order to be used instead of midazolam, such as clonidine [8, 9], melatonin [10], dexmedetomidine [11], and ketamine alone [12] or in combination with midazolam [13]. Also, there are other nonpharmacological interventions, such as parental presence at induction of anesthesia [14, 15], the use of clowns, toys, and art therapy [16, 17], traditional or web-based informational and orientation programs [18, 19], and family-centered preoperative [20] and educational [21] preparation workshops, that can be used instead. Special mention should be made in parental presence at induction of anesthesia since it is a common practice in many countries although its benefits cannot be generalized internationally [22]. As Fortier et al. [23] noted, parental cultural variables such as ethnicity and language may influence their desire for being present at induction of anesthesia and consequently the effectiveness of the intervention. To our knowledge, there is no recent evidence concerning Greek parent's desire for being present at their child's induction of anesthesia.

Determining the factors that contribute to significant preoperative anxiety levels in pediatric patients and their parents could help specialists, including nurses, and hospitals to choose the most appropriate one among several alternative strategies for anxiety control. According to Ahmed et al. [24], children risk factors for increased preoperative anxiety include their age and temperament, previous medical experience, and parent-child relationship. Risk factors that relate to parents have been found to be—among others—the age of the child, female gender, and previous hospitalization of the child.

The primary endpoint of this study is the identification of specific characteristics of both parents and children, such as education level, gender, child's age, previous hospitalization, and others in Greek population that may influence their level of preoperative anxiety. Secondary endpoints include the desire of parents to be present at their child's induction in anesthesia and the correlation between parent's and children's level of anxiety.

## 2. Materials and Methods

The study took place in University General Hospital of Alexandroupolis, located in the North-Eastern region of Greece, after the approval of Hospital's Board Committee and the informed consent of participants (parents). The study population consisted of 128 consecutive children (18 girls; 110 boys) with a median age of 5 years (range: 1–14 years; mean age of 5.61 ± 3.65 years) who were scheduled for elective minor surgery during 2014–2016 and their parents. The most frequent operations were phimosis, hypospadias, inguinal hernia, hydrocele, and undescended testicle repair under general anesthesia. Exclusion criteria were children's developmental delay, children that were not escorted by their parents, and families with a poor understanding of Greek language.

The collected demographic data included age and sex of the child, birth order, number of children in the family, previous hospitalization, place of residence, age, gender, and educational level of the parent (where high level indicates university education, medium level secondary education, and low level primary education) and were obtained from the parent who accompanied the child to the operating theatre's waiting room. Furthermore, parents were asked what method of preparation would be more helpful in reducing their anxiety level with a standardized question. It is worth mentioning that premedication was not included as an option, as it was in anesthetist judgment if it was necessary.

To evaluate children's anxiety, we used the Modified Yale Preoperative Anxiety Scale (m-YPAS) which was completed during the preoperative period by an experienced nurse. The m-YPAS is an observational behavioral checklist developed by Kain et al. [25] to measure the state anxiety of children. It contains twenty-two items divided into five categories: activity, vocalization, emotional expressivity, the state of arousal, and use of parents. Each category receives a score on a scale of four (six for vocalization) with higher scores indicating a higher level of anxiety. This scale is used widely in the medical literature and has a good-to-excellent reliability and validity for measuring children's preoperative anxiety [25].

The Spielberger State-Trait Anxiety Inventory (STAI) [26] was used to evaluate parent's anxiety. This is a self-report anxiety behavioral instrument which consists of two separate twenty-item subscales. The first—STAI-trait subscale—measures baseline anxiety in adults while the second subscale—STAI-state—measures situational anxiety. STAI is scored is based on 4-point Likert scale (1 = almost never to 4 = almost always) with a total score of 20–80 in each subscale. Higher scores indicate a higher level of anxiety.

### 2.1. Statistical Analysis

Statistical analysis of the data was performed using the Statistical Package for the Social Sciences (SPSS), version 19.0 (SPSS, Inc., Chicago, IL, USA). The Kolmogorov–Smirnov test for normality was performed. m-YPAS and STAI scores, which were normally distributed, were expressed as the mean ± standard deviation (SD), while categorical variables were expressed as frequencies (and percentage). Student's *t*-test and one-way analysis of variance (ANOVA) were used to determine differences in anxiety scores between two or more groups of participants, respectively; post hoc analyses were performed using Turkey's test. The chi-square test was used to evaluate any potential association between categorical variables. Pearson's r correlation coefficient was used to determine the association between m-YPAS and STAI scores. To assess the independent effect of demographic characteristics on m-YPAS and STAI scores, multivariate stepwise linear regression models were, respectively, constructed.

## 3. Results

A total of 128 children with their parents participated in this study. Demographic data and the results of measuring preoperative anxiety of study participants are shown in Table 1. The internal consistency of all three questionnaires was very high (Cronbach *α* coefficient was 0.73 for m-YPAS, 0.79 for STAI-state, and 0.82 for STAI-trait). Statistically significant positive correlations were observed between m-YPAS and STAI-state anxiety scores (*r*=0.493, *p* < 0.001), m-YPAS and STAI-trait anxiety scores (*r*=0.286, *p*=0.001), and STAI-state and STAI-trait anxiety scores (*r*=0.303, *p*=0.001). STAI-state anxiety score was statistically significantly higher than STAI-trait anxiety score (49.66 ± 6.19 versus 47.02 ± 4.53; *p* < 0.001).

Table 2 demonstrates the anxiety levels in relation to demographic characteristics. In particular, boy's parents seem to be less anxious (*p*=0.004) than girl's parents while sex does not affect how anxious the children are (*p*=0.198). Parents with younger children (<5) are displaying a higher level of anxiety than those having older children (*p*=0.001). The age of the child does not affect how anxious it is (*p*=0.660). As far as the number of children in the family and the birth order of the child are concerned, they do not influence the level of parental anxiety (*p*=0.844  and  *p*=0.669, resp.); however, patients that were the only child in their families have significantly more anxiety than those who have siblings (*p*=0.096). It is interesting that the previous hospitalization has no effect on parent's level of anxiety (*p*=0.512), while a tendency towards higher anxiety in children with previous hospitalization was observed (*p*=0.051). As it was expected, children that received premedication had significantly less anxiety than those that did not (*p*=0.001). Moreover, parents who knew that their children received midazolam were significantly less anxious, according to the STAI-state scale, than parents whose children did not get any premedication (*p*=0.001). Finally, families who live in rural areas demonstrated a greater degree of anxiety about the forthcoming operation than urban residents (parents: *p* < 0.001; children: *p*=0.009).

In addition, as it is shown in Table 2, mothers have more anxiety than fathers (*p*=0.019), and younger parents (*p*=0.015) are significantly more anxious than the older ones. Moreover, high- and low-educated parents have more anxiety (*p*=0.007) compared to those who have an average level of education. Finally, high STAI-trait anxiety status (>47, median value) was associated with higher STAI-state anxiety levels (*p* < 0.001) and higher children's anxiety levels (*p*=0.001) while STAI-state anxiety status (>50, median value) was associated with higher children's anxiety levels (*p* < 0.001).

Multivariate linear regression analysis disclosed that (i) no premedication (*p*=0.007), girls (*p*=0.008), child's age < 5 years (*p*=0.024), living in rural areas (*p* < 0.001), mothers (*p*=0.046), low or high education level of the parent (*p*=0.031), and high STAI-trait anxiety levels (*p*=0.003) remained statistically significant independent determinants of high STAI-state anxiety scores, which explain the 5.0%, 4.7%, 2.7%, 15.2%, 2.4%, 7.7%, and 8,4% of their variation, respectively, and (ii) high STAI-state anxiety status (*p* < 0.001), no premedication (*p*=0.014), previous hospitalization (*p*=0.019), and being the only child in the family (*p*=0.045) remained statistically significant independent determinants of high m-YPAS anxiety scores, which explain the 17.4%, 3.4%, 2.8%, and 2.5% of their variation, respectively (Table 3).

A total percentage of 74.2% of parents would like to be present at induction in anesthesia of their child. Other strategies that could help parents control their anxiety, according to their opinion, include a more detailed preoperative interview and informational program (69.5%), behavioral and psycho-educational interventions such as development of coping skills, operating room tour, role playing, exposure to anesthetic equipment (35.2%), clowns, toys, and distraction activities (32.8%), and alternative medicine strategies like hypnosis, acupuncture, and music therapy (10.2%).

## 4. Discussion

Preoperative anxiety reduction in pediatric patients undergoing elective surgery still constitutes a challenging issue. Given the fact that, to the best of our knowledge, there are very few studies on children's and parent's preoperative anxiety in Greek population and taking into consideration the different practices which are adopted by the various hospitals, this study attempted to identify specific factors of preoperative anxiety so that targeted policies of reducing preoperative anxiety could be planned and implemented.

The sample was composed of 128 children (1–14 years of age) who had to undergo minor surgery. Apart from the children that were excluded according to the criteria that were set, no one refused to participate in the study. We believe the reason is that our hospital is a tertiary referral centre and most of the patients are specifically referred to it and are familiar with the fact that research is part of our duties.

According to the findings of the present study, the factors that clearly related to high anxiety levels in children were previous hospitalization, anxious parents, being the only child in a family, and the fact that they have not been premedicated. As argued in other studies as well, previous hospitalization [27] and high parental anxiety levels [28] increase children's anxiety. The association between premedication and reduced anxiety has been indicated by several other studies as well [4, 29]. Another finding of our analysis is the fact that being the only child in a family contributes to higher level of this child's anxiety. A possible reason for that could be that these patients have not got the opportunity to share their feelings with a sibling, but further research is needed in this matter.

In addition to these, it seems that parent's anxiety is influenced by children's age and higher stress characterizes parents with younger children (less than five years old) probably due to the difficulty to communicate with them. Also, mothers have more anxiety, a finding that agrees with similar conclusions in other studies [15] while it seems that there is a positive correlation between parent's age and their level of anxiety, with the younger ones having more anxiety than the older ones. Families who are living in rural areas are characterized by a higher level of anxiety compared with cities inhabitants. A potential reason for this difference could be the additional difficulties they are facing, such as longer distances to commute and the need of accommodation because of the surgery, the expenses, and so on. Parent's higher educational background seems to correlate positively with their level of anxiety, with one possible explanation being their more detailed knowledge of the surgery's potential risks. On the other hand, it is interesting that parents with a low educational background have a high level of anxiety as well, which can be justified by their lack of information.

As it is found by multivariate analysis in the present study and is indicated in others [30, 31], parental anxiety influences their children negatively, as it was found to be the strongest independent risk factor (beta-coefficient = 0.366) for children's preoperative anxiety levels. Thus, efforts must be taken to decrease parent's preoperative stress with behavioral or other interventions. There is a need for further research on these actions, targeting parent's anxiety, on Greek population. More information about the perioperative period, psychoeducational and other supporting programs for parents, could improve their attitude which would further facilitate reducing their children's anxiety.

Results of the present study depict a strong desire of parents to be present at induction in anesthesia. Although parental presence does not seem to be beneficial if used as a routine in reducing preoperative anxiety in children compared to sedative premedication or other distractive techniques [32], it does increase parental satisfaction with anesthesia process and the overall functioning of the hospital [14]. Further research is needed in order to evaluate specific parental characteristics that may impact desire and motivation for parental presence during induction of anesthesia.

## 5. Conclusions

By the findings of this study, useful conclusions have been obtained related to factors which characterized pediatric patients and their parents with high preoperative anxiety levels. Previous hospitalization, being the only child in a family, and parental anxiety level were found to be the most significant risk factors for increased preoperative anxiety in children. Parental characteristics that influence their level of anxiety include child's and parent's age, female gender, high- and low-educational background, living in rural areas, and their anxiety as a trait. Identifying those characteristics could help appropriate anxiety control interventions to be developed and implemented. According to parent's opinion, being present during induction of anesthesia may contribute in controlling their anxiety levels while other strategies include a more detailed preoperative interview and informational programs, behavioral and psychoeducational interventions, and various distraction activities.

## Figures and Tables

**Table 1:** Demographics of the study participants and their parents.

	Number of patients	Percentage (%)
Sex		
Boys	110	85.9
Girls	18	14.1
Age (years; mean ± SD)	5.61 ± 3.64	
Number of children in the family		
1 child	45	35.2
2 children	53	41.4
More than 2 children	30	23.4
Birth order		
1st	60	46.9
2nd	54	42.2
3rd or higher	14	10.9
Place of residence		
Rural	98	76.6
Urban	30	23.4
Previous hospitalization		
No	85	66.4
Yes	43	33.6
Parents' gender		
Males	21	16.4
Females	107	83.6
Age of the parent (years; mean ± SD)	34.20 ± 5.91	
Parents' education level		
Low	27	21.1
Medium	63	49.2
High	38	29.7
Premedication		
No	64	50.0
Yes	64	50.0
Anxiety of the child in the waiting room (m-YPAS; mean ± SD)	38.52 ± 9.59	
Trait anxiety of the parent (STAI Y-2; mean ± SD)	47.02 ± 4.53	
State anxiety of the parent (STAI Y-1; mean ± SD)	49.66 ± 6.19	

**Table 2:** State anxiety of the parent (STAI Y-1) in relation to demographics of the study participants and their parents.

	STAI Y-1	*p* value	m-YPAS	*p* value
Sex		0.004		0.198
Boys	49.03 ± 6.08		38.07 ± 9.67	
Girls	53.50 ± 5.59		41.22 ± 8.90	
Age		0.001		0.660
≤5 years	51.38 ± 5.44		38.14 ± 9.35	
>5 years	47.94 ± 6.46		38.89 ± 9.89	
Number of children in the family		0.844		0.096
1 child	49.71 ± 7.55		40.29 ± 12.60	
2 children	49.92 ± 5.31		38.75 ± 7.93	
More than 2 children	49.10 ± 5.52		35.43 ± 5.86	
Birth order		0.669		0.785
1st	49.17 ± 7.01		38.43 ± 10.70	
2nd	49.96 ± 5.83		39.00 ± 9.15	
3rd or higher	50.57 ± 3.23		37.00 ± 5.87	
Place of residence		<0.001		0.009
Urban	48.33 ± 5.40		37.14 ± 8.86	
Rural	54.00 ± 6.71		43.00 ± 10.64	
Previous hospitalization		0.512		0.051
No	49.40 ± 6.46		37.40 ± 9.99	
Yes	50.16 ± 5.66		40.72 ± 8.41	
Parents' gender		0.019		0.102
Males	46.76 ± 4.94		35.38 ± 10.47	
Females	50.22 ± 6.27		39.13 ± 9.34	
Age of the parent		0.015		0.270
≤35 years	50.98 ± 6.59		39.45 ± 9.97	
>35 years	48.33 ± 5.50		37.58 ± 9.18	
Parents' education level		0.007		0.358
Low	51.56 ± 7.06		36.30 ± 7.61	
Medium	47.94 ± 5.86		38.75 ± 9.36	
High	51.16 ± 5.39		39.71 ± 9.49	
Premedication		0.001		0.001
No	51.52 ± 6.22		41.23 ± 10.21	
Yes	47.80 ± 5.62		35.80 ± 8.14	
STAI-trait anxiety status		<0.001		0.001
Low (≤median value)	47.34 ± 5.04		35.68 ± 7.86	
High (>median value)	52.05 ± 6.39		41.44 ± 10.37	
STAI-state anxiety status				<0.001
Low (≤median value)	—		34.89 ± 8.71	
High (>median value)	—		42.90 ± 8.80	

**Table 3:** Multivariate analysis for STAI-state and m-YPAS anxiety scores.

	Unstandardized *b* coefficient	Standard error	Unstandardized beta coefficient	*t*	*p* value
STAI-state anxiety					
Constant	31.926	2.888	—	11.055	<0.001
Girls	3.471	1.311	0.196	2.647	0.008
Child's age >5 years	2.133	0.931	0.173	2.291	0.024
Living in rural areas	4.209	1.071	0.289	3.930	<0.001
Mothers	2.414	1.198	0.145	2.015	0.046
Low or high education level	2.044	0.939	0.166	2.178	0.031
Without premedication	2.410	0.878	0.195	2.744	0.007
High STAI-trait anxiety score	2.768	0.919	0.224	3.013	0.003

m-YPAS					

Constant	31.059	1.420	—	21.873	<0.001
High STAI-state anxiety score	7.029	1.537	0.366	4.572	<0.001
Without premedication	3.806	1.532	0.199	2.484	0.014
Previous hospitalization	3.739	1.579	0.185	2.368	0.019
Only child in the family	3.163	1.560	0.158	2.028	0.045

## Data Availability

The data used to support the findings of this study are available from the corresponding author upon request.
